# Historicizing Modern Slavery: Free-Grown Sugar as an Ethics-Driven Market Category in Nineteenth-Century Britain

**DOI:** 10.1007/s10551-019-04318-1

**Published:** 2019-10-28

**Authors:** Andrew Smith, Jennifer Johns

**Affiliations:** 1 University of Liverpool Management School, Chatham St, Liverpool, L69 7ZH UK; 2 Department of Management, University of Bristol, 12A Priory Rd, Bristol, BS8 1TU UK

**Keywords:** Slavery, Market categories, Consumption ethics

## Abstract

The modern slavery literature engages with history in an extremely limited fashion. Our paper demonstrates to the utility of historical research to modern slavery researchers by explaining the rise and fall of the ethics-driven market category of “free-grown sugar” in nineteenth-century Britain. In the first decades of the century, the market category of “free-grown sugar” enabled consumers who were opposed to slavery to pay a premium for a more ethical product. After circa 1840, this market category disappeared, even though considerable quantities of slave-grown sugar continued to arrive into the UK. We explain the disappearance of the market category. Our paper contributes to the on-going debates about slavery in management by historicizing and thus problematizing the concept of “slavery”. The paper challenges those modern slavery scholars who argue that lack of consumer knowledge about product provenance is the main barrier to the elimination of slavery from today’s international supply chains. The historical research presented in this paper suggests that consumer indifference, rather than simply ignorance, may be the more fundamental problem. The paper challenges the optimistic historical metanarrative that pervades much of the research on ethical consumption. It highlights the fragility of ethics-driven market categories, offering lessons for researchers and practitioners seeking to tackle modern slavery.

## Introduction

Over the last 15 years, social activists have succeeded in raising awareness of the existence of slavery and in forcing governments and firms into tackling this problem (Murphy 2015). The victims of so-called “modern slavery” work in agriculture, construction, quarries, brothels, homes, and other places. Approximately 30 million people in the world today can be reasonably described as enslaved (Bales 2000; ILO 2012). The products of their labour often end up in the supply chains of multinational firms. Management academics have recently begun to participate in the on-going academic conversations about modern slavery (e.g., Crane 2013; Crane et al. 2017). Unfortunately, the management research on modern slavery is largely ahistorical as it ignores the parallels and continuities with historical forms of slavery. Our paper uses the British sugar market in the era in which slavery was gradually being suppressed in Western countries to refine our understanding of the relationship between shifting ethical norms, consumption ethics, and market categories. We do so in a historical cognizant history-to-theory study (Kipping and Üsdiken 2014) that illuminates the relationship between shifting attitudes towards slavery and the consumption of the products of slaves.

To help us to understand the historical phenomena discussed in our paper, we draw on business ethics literature to develop the concept of the *ethics*-*driven market category*. We then apply this concept to the British sugar trade in the historical period in which the legitimacy of slavery was being contested by activists. In the 1790 s, a small group of British entrepreneurs with ties to the East India Company created a new market category, “free-grown sugar”. This market category appealed to British consumers who supported the new anti-slavery movement and who did not want to support the institution of slavery by purchasing sugar from the West Indies. From the 1790s to the 1830s, UK retailers offered consumers the choice between slave-produced and non-slave sugar products. During this period, significant numbers of British consumers paid a premium for sugar that was marketed as being produced without slave labour. After 1840, the ethics-driven market category of free-grown sugar vanished from Britain, even though the country continued to import vast quantities of slave-produced sugar, especially after controversial tariff modifications in 1846 the made it easier to import sugar from jurisdictions such as Cuba and Brazil in which was slavery was legal until the 1880s.

Our paper contributes to the on-going debates in management about slavery by historicizing and thus problematizing the concept of “slavery” and showing that its boundaries have long been contested. The paper challenges those modern slavery scholars who argue that lack of consumer knowledge about product provenance is the main barrier to the elimination of slavery from international supply chains by arguing that consumer indifference, rather than simply ignorance, may be the more fundamental problem. The paper integrates factors such as race and ethnicity into our understanding of how consumers think about slavery, which implies that modern slavery scholars should examine these variables. The paper challenges the optimistic historical metanarrative that pervades much of the research on ethical consumption and thus reminds modern slavery researchers, of the utility of incorporating historical research into their analysis of modern forms of slavery.

## Literature Review

### Research on Modern Slavery

Around the year 2007, the term *modern slavery* came into widespread use by academics concerned with the continued existence of various forms of highly unfree labour (Bhoola 2007; Davidson 2015; Craig et al. 2019). Prior to 2007, the term “modern slavery” was used rarely and is found primarily in documents about unfree labour in Africa and the Arabian Peninsula (Ross 1925). The coining of the term “modern slavery” has helped to focus the minds of practitioners and academics on this important issue. Papers on modern slavery have appeared in leading social-scientific journals in fields such as sociology and political economy (LeBaron and Ayers 2013; LeBaron 2014a, b Strauss and McGrath 2017). Academics have also published important books on the topic of modern slavery (Bales 2000; Kara 2017; Davidson 2015; Scarpa 2011).

There is no universally accepted definition of “modern slavery”. The first definition of slavery in an international agreement appeared in the Slavery, Servitude, Forced Labour and Similar Institutions and Practices of Convention of 1926, which defined slavery as “the status or condition of a person over whom any or all of the powers attaching to the right of ownership are exercised” (League of Nations 1926). Since 2007, the term “modern slavery” has become widespread in debates around most forms of severe exploitation (Craig et al. 2019). The UK and Australia have passed legislation with “modern slavery” in the title (passed in 2015 and 2018, respectively). Bhoola (2007) notes that the practices encompassed by the term “modern slavery” cover traditional slavery; institutions and practices similar to slavery, such as debt bondage, serfdom and forced marriage; and forced labour. Others have expanded the concept of modern slavery to include the most severe forms of economic exploitation (Bales 2007; Craig et al. 2019). Davidson (2010, p. 257) argues that it is legitimate for slavery to be defined flexibly and through a series of subjective judgements about where “appropriate” exploitation ends and “inappropriate” exploitation starts.

The academic literature on modern slavery consists, overwhelmingly, of books and papers that were published by scholars who work outside of management schools. Cooke (2003, p. 1895) argued persuasively that there is a “denial of slavery in management studies”. As Crane (2013) observed in his path-breaking paper on modern slavery, the absence of work on slavery in management research is problematic because it ignores the role of companies and managers in one of the most acute abuses of human rights in the contemporary economy. Crane called for management scholars to engage with the topic on the grounds that “Management research can, however, play an important part in explaining the persistence of slavery in the face of rules, norms and practices to the contrary” (Crane 2013, p. 49).

Since Crane’s paper appeared in 2013, six papers on modern slavery have appeared in management journals. Writing in a supply chain management journal, Gold et al. (2015) discuss how efforts to end modern slavery will impact supply chain management. Their paper connects research on slavery from other disciplines to the literature in supply chain management on management tools and indicator systems. They argue that the development of more effective indicators aimed at detecting the presence of slave labour in the supply chain must consider the specific social and cultural context of supply regions. New (2015) discusses forced labour in the supply chain and explains why conventional CSR theory may be incapable of addressing the problem of modern slavery. Building on Crane, New argues that the standard initiatives of anti-modern slavery CSR are themselves part of the enabling mechanisms that allow modern slavery to persist.

Crane returned to the subject of modern slavery in a co-authored paper that examines forced labour in UK domestic supply chains, offering a cross-industry comparison of the regulatory gaps surrounding forced labour in the UK (Crane et al. 2017). Drawing on political science research on modern slavery (e.g., LeBaron 2014a; LeBaron and Rühmkorf 2017), Crane et al. argue that addressing governance gaps around forced labour requires new thinking about how to design operative governance that is sensitive to local cultural contexts. Christ and Burritt (2018) examine modern slavery in an accounting journal paper that considers the recent efforts of the Australian policymakers to suppress modern slavery. They identify the “ignorance” of consumers about the provenance of products and the relative powerlessness of employee representatives as important barriers to the elimination of modern slavery in the supply chains of Australian firms. Their emphasis on consumer ignorance implies that consumers in Australia are unwittingly buying the products of slave labour and that such consumers would change their purchasing decisions if only they could tell which products contain value added by slaves. Christ and Burritt do not appear to have considered the possibility that moral indifference, as opposed to simple ignorance and powerlessness, characterizes how Australian consumers and workers respond to slavery in distant countries.

Stevenson and Cole (2018) analyse the information disclosed by firms in response to the UK legislation on transparency in supply chains (the Modern Slavery Act). They suggest that managers need to acknowledge that the practices currently employed to detect and remediate other social issues may not apply to modern slavery (p. 94). Stringer and Michailova (2018, p. 1), publishing in an international business journal, suggest three factors that explain how modern slavery can occur in global value chains; their complexity and the resulting challenges for governance, the business case for slavery and the conditions that enable modern slavery. Firms create supply chain complexity through strategies that include outsourcing to legally separate firms, which makes it harder for outsiders to detect the use of slave labour.

Unfortunately, the extant research in management on modern slavery is largely ahistorical. New (2015) suggests that there is little scholars interested in modern slavery could learn from historical research on slavery and supply chains when he suggests that modern slavery is radically different “from previous models of servitude, such as possessive (chattel) slavery in ante-bellum North America or in the ancient world, which included legally sanctioned ownership of people” (New 2015, p. 1). The ahistorical nature of the management research on modern slavery is problematic as it depicts modern slavery as isolated and essentially unconnected from the historical forms of slavery with which we are most familiar, such as African chattel slavery (Craig et al. 2019). As Craig et al. rightly argue, obscuring the continuities and similarities between “old slavery” and “modern slavery” is unjustifiable because many of the commodities, business practices, and regions of the world that were closely connected to “old slavery” are those that are most closely associated with “modern slavery”. Management research on modern slavery has overlooked the historical continuities between contemporary management practices, such as cost accounting, and slavery (Rosenthal 2018). Another downside of the ahistorical nature of the management research on modern slavery is that it closes off an important avenue of research, historical research methods.

The prevailing ways of thinking about the history of “slavery as an institution” distort our ability to recognize slavery in the present (Miller 2012, p. 1). The common perception that slavery was permanently abolished in the nineteenth century has contributed to the inability of many observers today to detect the presence of slavery, even when the signs of slavery are visible (Murphy 2015, p. 391). The associated habits of thought have discouraged the production of academic studies that connect and/or compare historic and contemporary slavery (exceptions include Smith 2007; Stetson 2007; Quirk 2012). Modern slavery has taken on new forms, but, as Quirk (2012) argues, it must be understood as an extension and/or reconfiguration of historical slavery rather than something entirely new. Our paper explicitly seeks to connect “old” and “contemporary” forms of slavery via a theoretical frame centred on the concept of the ethics-driven market category.

### Ethical Consumption

Tackling modern slavery necessitates consideration of the interaction between a range of different actors. In consequence, much attention has been paid to the purchasing decisions of consumers due to the observed increase in visibility of so-called ‘ethical consumers’ concerned with the conditions of the workers producing their food and consumer goods (Harrison et al. 2005). Ethical consumption is the behaviour of ethically minded consumers who feel accountable for the environment and towards society (Freestone and McGoldrick 2008; Harrison et al. 2005). Researchers have discussed the recent emergence of a new type of consumer, the “ethical consumer,” whose purchasing decisions are informed by a sense of responsibility towards the environment and/or to society. Such consumers, according to this literature, express their values through ethical consumption and purchasing (or boycotting) behaviour (De Pelsmacker et al. 2005; Shaw and Shiu 2002; Carrington et al. 2010). According to these scholars, the ethical concerns that shape consumer behaviour include sustainability, fair trade, animal welfare, environmental/green issues, and workers’ rights (including modern slavery). The extant research strongly associates ethical consumption with the contemporary youth, implying that this age cohort is more attuned to ethical issues than previous generations (Bucic et al. 2012).

Sebastinani et al. (2013) has identified two blind spots in the existing research on ethical consumption that require more attention. First, the existing research ethical consumption focuses too much on demand and not enough on supply-side considerations, such as the motives of the entrepreneurs who supply consumers with ethical products. Second, the existing research has given us an imperfect understanding of the ‘attitude-behaviour gap’ or ‘ethical purchasing gap’ (Boulstridge and Carrigan 2000; Nicholls and Lee 2006; Auger and Devinney 2007; Caruana et al. 2016). There is often a disparity between what consumers say about the importance of ethical issues and the preferences revealed in their actual purchasing decisions (Auger and Devinney 2007; Carrington et al. 2010; Nicholls and Lee 2006). Sebastinani et al. (2013) suggests that much more research on these gaps is required.

### Market Categories

The primary focus of this paper is to address debates about slavery and ethical consumption. In dealing with the issue of slavery and ethical consumption, it is helpful to draw on the conceptual work on market categories (Lounsbury and Rao 2004; Benner 2007; Vergne and Wry 2014). Market categories help individuals to evaluate “organizations and their products.” Categories, which are “cognitive shortcuts” (Hsu and Grodal 2015, p. 55), inform the expectations of market participants (Durand and Paolella 2013). Categories research stresses the importance of prototypes and shows that market participants generally punish producers who deviate too much from the category’s prototype (Hsu et al. 2009; Leung and Sharkey 2014). Categories researchers have also noted that categories vary in their degree of *fuzziness* (Kovács and Hannan 2010): when categories are *crisp* (i.e., have clear boundaries), actors are more likely to be punished for deviating from categories than if the relevant categories are fuzzy. Durand and Khaire (2017) firmly distinguish *category emergence* from *category creation*. Category emergence occurs when a market’s existing classification system is ill-equipped to deal with material innovations (e.g., Navis and Glynn 2010). In contrast, category creation involves the development of a new category via the creation of a mental boundary around a subset of an existing category (Khaire and Wadhwani 2010). Free-labour sugar, the category whose rise and fall is charted in this paper, is an example of a created category rather than one that emerged.

In a paper that attempts to bridge the business ethics and market categories literatures, Arjaliès and Durand (2019) observe that some created categories are morally differentiated subsets of larger categories. For instance, “FairTrade chocolate” is a subset of the market category of chocolate, one that is purchased by people who feel they have an ethical obligation towards cocoa producers. This ethics-driven market category helps the individual consumer to associate the product attributes that are important to her with particular products, reducing ambiguity and thus cognitive load. Similarly, the category of cruelty-free cosmetics appeal to those concerned with animal welfare. Such market categories come into existence because of a prior cultural shift that resulted in the development of a segment of the consumer population that is willing to pay a premium for goods that are perceived as ethically superior and the advent of entrepreneurs who help to create such market categories. Until very recently, the literature on market categories did not pay attention to how changing conceptions of ethics and morality influence the evolution of market categories. In a study of the rise of the Socially Responsible Investment (SRI) fund in France in the period 1997 to 2017, Arjaliès and Durand (2019) have develop a phase model for understanding how increased awareness of an ethical issue can result in the development of a market category. In the first phase of their model, “judgment silence” the ethical issues related to a product go undiscussed by market participants. In the second phase, “turmoil and judgement questioning,” the ethics of the product are intensively debated, which leads to the emergence of a category that includes “normative attributes”. In the final phase, “stability and judgment inclusion” the market category is firmly established.

In our view, Arjaliès and Durand’s (2019) linear model overlooks several important considerations. First, because the final phase in their model is the successful establishment of a product category that includes moral attributes (what we would call an ethics-driven market category), the model implies that there will not be a return to silencing. In our view, it is entirely possible for a market category that develops due to shifts in the prevailing thinking about a moral issue to subsequently disappear following yet another change in how people think about that issue. We thus conceptualize ethics-driven market categories as more fragile than the model of Arjaliès and Durand allows. Moreover, while Arjaliès and Durand briefly mention the political context in which SRI funds became an important part of the French financial system, their paper does not consider, in-depth, the socio-cultural context in which the SRI category emerged. As both Kipping and Üsdiken (2014) and McLaren and Durepos (2015) argue, management scholars frequently ignore historical context in their case studies. Although Arjaliès and Durand (2019) pay somewhat more attention to socio-cultural context than many other management case studies, the level of contextual analysis in their paper is low compared to that which typically appears in historical papers. A tendency to downplay the importance of local context is a pronounced feature of the existing categories research (Grodal and Kahl 2017). In contrast, our paper presents extensive information about the context of the market category of free labour and then uses the research of cultural historians to explain the rise and fall of this ethics-driven market category. Our analysis of the relationship between category of free-labour sugar and its historical context reveals that problems with the linear model of Arjaliès and Durand’s (2019).

### Historical Metanarratives

In addition to critiquing the linear model of Arjaliès and Durand, our paper challenges the historical metanarratives that many business ethics researchers use to understand the world. A historical metanarrative is a story about human history that allow one to make sense of data points by constructing narratives that cover events in a particular time and place. Individuals use historical metanarratives to interpret data so as to construct more meaningful historical narratives and to make predictions (Robinson and Hawpe 1986; Shucksmith et al. 2011; Butters 2017). Historical metanarratives can shape the thinking of a researcher without her becoming conscious of it. Since the Enlightenment of the eighteenth century, one of the most pervasive historical metanarratives in Western culture has been a linear-progressive metanarrative (Spadafora 1990; O’Brien 2001). Linear-progressive metanarratives posit that each generation will be better off than its predecessors in material wealth, technological prowess, and, crucially, level of moral development.

The leading academic proponents of linear-progressive historical narratives include Singer (2011) and Pinker (2011, 2018). Singer (2011), who is arguably the most influential living moral philosopher, regards the expansion of the “circle of ethical concern” that began during the Enlightenment as a key driver of moral progress. He argues that individuals have always sought to act ethically towards their kinfolk and neighbours, but that the expression of concern for the welfare of distant individuals is a uniquely modern phenomenon. Singer’s metanarrative allows him to link together a wide variety of phenomena ranging from the anti-slavery movement of the nineteenth century to twenty-first century animal rights campaigns in a single narrative arc. Singer’s influential “expanding circle” concept is a clear example of the linear-progressive historical metanarrative discussed above. Writing in a similar vein, Pinker (2011, p. 2017) argues that human beings have become kinder and more empathetic with each passing generation. Pinker causally connects the “humanitarian revolution” of the last three centuries to the emergence of stable governments, democratization, the commercialization of societies, and the growing influence of the eighteenth-century Enlightenment philosophers he admires. The key point here is that both Singer and Pinker view the world through a linear-progressive historical metanarrative that has optimistic implications. As we show below, this historical metanarrative informs and, in our view, distorts how many scholars understand ethical consumption.

The literature in management on consumer ethics is pervaded by a linear historical metanarrative that holds that there is a natural tendency for consumers to become more interested in ethical issues with the passage of each generation. According to this metanarrative, as people become wealthier and thus more empathetic, their behaviour comes to be influenced by ethical concerns. Their purchases come to be influenced by moral considerations related to matters such as global warming, FairTrade, and modern slavery (Barnett et al. 2005). In other words, they become more willing than their ancestors to pay a premium for more expensive ethical products (Boulstridge and Carrigan 2000; Bray et al. 2011; Carrigan and Attalla 2001; De Pelsmacker et al. 2005; Mohr et al. 2001). Some scholars of consumer ethics use the term “postmaterialist” (Inglehart 1977) to describe the ever-growing number of consumers who are prioritize ethical concerns or their own material self-interest (e.g., Promislo et al. 2017). Inglehart’s theory of history holds that people who have grown up in eras of affluence will prioritize self-expression and the pursuit of social and environmental justice. The optimistic implication of this linear-progressive historical metanarrative is that one would expect each generation of consumers to be more humane, more ethically aware, and more likely to boycott unethical products than the generation that went before it.

We see the influence of this linear-progressive historical metanarrative in the extensive research on the rise of CSR. Business ethics academics generally regard this phenomenon as a very recent development and most of the narratives about the rise of CSR focus on the period since circa 1990 and on countries with high GDP per capita (Burke et al. 2014; Carfagna et al. 2014; Moraes et al. 2017). Similarly, as Newholm et al. (2015) have noted, the “rise of the ethical consumer” is commonly conceptualized by marketing academics as having taken place relatively recently (i.e., in the late twentieth century) and only in the world’s wealthiest societies. These authors suggest that consumers’ attention to ethics intensifies as GDP per capita increases. The underlying causal mechanism apparently envisioned by these scholars depicts consumer ethics as a type of luxury good: once incomes climb beyond bare subsistence, consumers’ definitions of utility will inevitably broaden to include ethical considerations, thereby prompting changes in firm strategy. In keeping with the linear-progressive historical metanarrative that is so prevalent in management research, business ethics and marketing scholars have argued that so-called “Millennials” are more ethically conscious than previous generations. They argue that firms will have to adjust their strategies as a result (Smith 2011; Bucic et al. 2012; VanMeter et al. 2013; Culiberg and Mihelič 2016; Weber and Urick 2017). The implication of this research stream is that each generation will be more ethical than the one that went before. Researchers have also asserted that as poor countries become wealthy, their consumers will naturally acquire a greater desire to make ethical purchasing decisions (Guarin and Knorringa 2014; Yin et al. 2018). Again, this prediction is informed by a linear-progressive historical metanarrative similar to those of Singer and Pinker, albeit one that is never explicitly acknowledged and/or defended.

Trentmann (2007) is a rare example of a paper on the history of consumer ethics that challenges the dominant “linear progressive” metanarrative. Trentmann (2007) argues that the emergence of the ethical consumer has been a decidedly non-linear process marked by frequent periods of retrogression in which the level of interest in ethical issues revealed in consumer behaviour falls rather than increases. In Trentmann’s schema, social reformers seek to *moralize* the consumption of a given commodity that previously had been regarded as morally unproblematic. According to Trentmann, it is possible for a commodity that has been moralized to be subsequently *demoralized* (i.e., for consumers to stop caring about the ethics of the business practices associated with the commodity). In our case study, we document a process of moralisation and demoralization.

### Methodology and Data

In an important work on historical organizational studies, Maclean et al. (2016, Fig. 1), identify four types of historical research within organization studies: *evaluating, explicating, conceptualizing,* and *narrating.* Using a distinction first made by Fogel and Elton (1983), Maclean et al. characterize the first two types of historical research as classically social scientific, whilst the latter two as narrative. The approach to historical research adopted in this paper corresponds to the conceptualizing mode they discuss. Maclean et al. observe (2016, p. 614) that the value to organization studies of “History As Conceptualizing… lies in generating new theoretical constructs.” They describe David’s highly-cited (1985) paper on the historical origins of the QWERTY keyboard, which helped to establish the concept of “path dependence,” as an example of the use of historical material to create a new concept. As Maclean et al. observe, historical research of this type is often done using grounded theory (Glaser and Strauss 1967), which means that the researcher generates insights, and thus new concepts, from an inductive process that involves the review of large quantities of data.

Maclean et al. argue that high-quality research in historical organization studies has the following hallmarks: *dual integrity*, *pluralistic understanding*, *representative truth*, *context sensitivity*, and *theoretical fluency*. We have sought to adhere to all of these principles here. Dual integrity means that both historians and organization studies scholars would respect a paper if they encountered it. We share their view that dual integrity is important in historical organizational studies because it respects the fundamental values of the discipline of history. According to Maclean et al., pluralistic understanding, which denotes a high degree of openness to alternatives ways of interpreting the phenomenon being studied, is important when conducting historical research aimed at producing a new category because it stimulates creative thinking. Representational truth, which means that there is congruence between evidence, logic, and interpretation, is essential for a researcher’s findings to be regarded as plausible. Context sensitivity (being aware of differences between different societies and periods) is useful in identifying the role of contingency, while theoretical fluency (being familiar with the on-going theoretical debates in the field of organization studies) is crucial in making and producing new constructs.

This paper draws on the work of Maclean et al. (2016). Our approach was also informed by insights presented by Stutz and Sachs (2018). Dissatisfaction with the limitations of the positivist approaches (Eisenhardt 1989; Eisenhardt et al. 2016) and constructionist approaches (Gioia et al. 2013) to qualitative research in management prompted Stutz and Sachs to develop a new approach called the *reflexive historical case study* (RHCS). Their approach, which is informed by the work of the philosopher Hans-Georg Gadamer, is designed to allow researchers to contribute to the development of theory. Stutz and Sachs call on researchers to pay more attention to both their own social contexts and, crucially, the social and historical contexts of their case studies. Stutz and Sachs argue that the approach exemplified by Eisenhardt encourages researchers to unnecessarily “uproot” and decontextualize their case study research. We wholeheartedly agree with their view that case study research in management ought to pay more attention to political, social, and economic context.

In an important paper on historical embeddedness, Vaara and Lamberg (2016, p. 634) have argued that just as the phenomena they study are embedded in particular “socio-historical” contexts, academic researchers are also “embedded in socio-historical environments” that shape how they perceive reality. In conducting the abductive research presented in this paper, the authors were therefore conscious of their own preconceptions, biases, and limitations as twenty-first century researchers examining texts created by individuals with eighteenth- and nineteenth-century worldviews. As researchers who live and work in a highly secularized society (the contemporary British mainland), we were aware that understanding the worldview of the historical actors in our case study would involve the leaps of imagination necessary to understand the thinking of individuals of a more religious era. We were also conscious that our deep commitment to the issue of modern slavery, and our centre-left ideological scepticism about the social effects of unregulated market forces, might bias our interpretation of primary sources via confirmation bias. During our analytical process, we therefore took active steps to limit the extent to which these subjective biases might distort our data analysis. These steps are described below.

We have sought to adhere to all of the five principles identified by Maclean et al. (2016), particularly dual integrity, the very first of the five principles they list. The research presented in this paper was conducted using the business-historical methods described by Kipping et al. (2014), Lipartito (2014), and Wadhwani and Decker (2017). Business-historical researchers, like other historical researchers, clearly distinguish between primary and secondary sources. For such researchers, the term primary source denotes a document created at the time of the historical phenomenon being studied, ideally by an eyewitness (Lipartito 2014). In contrast, the term secondary source means, for a historian, a document written long after the phenomenon being described (Howell and Prevenier 2001, pp. 60–63; Langlois and Seignobos 2014). Historians distinguish various types of primary sources (e.g., diaries, correspondence, and newspapers). They also sub-divide secondary sources into peer-reviewed and non-peer reviewed. In business-historical research, the scholar first identifies a research question and then reviews the extant secondary sources on that topic. Today electronic databases such as Academic Search Complete are used to conduct comprehensive literature searches. Since some secondary sources are not yet available electronically, the review of the secondary literature about a topic often involves visits to specialist research libraries. Reading the secondary literature gives the researcher an understanding of both the current scholarly debates about a topic but also the topic’s chronology and the identities of the key individual and corporate actors. Formulating a detailed timeline and a list of the leading personalities involved in a historical phenomenon facilitates the researcher’s subsequent search for primary sources relevant to her topic.

At the start of this project, the authors used the *Academic Search Complete* database to produce a comprehensive list of all of the secondary sources that might be relevant to our project. (see Table 1 on historical databases consulted). Using the full-text search function of the *Historical Abstracts* database, we searched for papers that discussed both “sugar” and “slavery”, which produced 2924 results dating from 1958 to 2017. Of these, 2678 sources were peer-reviewed and 2646 were in English. Examination of these results revealed that most of these papers and books related to life on plantations in the Caribbean and did not discuss the sugar trade within the British Isles, our topic. We therefore further narrowed our search to publications that the creators of the database had coded as being about Great Britain. Delimiting our search in this way reduced the number of books and articles to 152, 150 of which were in English. We then identified which of these publications discussed the relationship between sugar and slavery in a sustained way as opposed to simply making a passing reference to both slavery and sugar, which left us with thirty secondary sources. The authors then secured access to all of these books and articles and read them, making notes. We supplemented this reading by re-reading books that are recognized classics in the study of slavery and abolitionism, such as *Capitalism and Slavery* (Williams 1944) which appeared too early to be captured by the *Historical Abstracts* search but which we knew from prior research projects. To get access to these texts, we visited research libraries in Liverpool, a port city with historic ties to the slave trade that is now a leading centre of academic research on slavery, historic and present-day.

**Table 1:** Historical databases consulted

Database and type	Description of database	Search terms used	Nature of material and number of relevant documents found	Purpose of analysing this material/nature of material was founds
Historical texts (scholarly source)	460,000 texts from invention of printing to 1914. Predominantly English language books from the British Library.	“Free-grown sugar”, “free-labour sugar” “Slave-grown sugar”	Books, book chapters and research summaries 24 results for free-grown sugar 76 results for slave-grown sugar	Analysis provided an overview of the key actors and processes involved in the market category formation and (re)silencing. We found a considerable number of documents that gave us the perspectives of policymakers, fewer that expressed the ideas of entrepreneurs, and very few that captured the voices of sugar consumers
House of commons parliamentary papers (historical source)	Keyword-searchable database of documents created by or for the British parliament	“Free-grown sugar”, “free-labour sugar” “Slave-grown sugar”	75 total results from 1833 to 1902. These took the form of records of speeches in parliament (50 results); reports of parliamentary committees (12 results); papers presented to parliament by civil servants (13 results)	The documents we found provide us with statistical data that allowed us to understand the important role of slaves in supplying the UK’s sugar needs up to the 1870s. Other materials provided us with insight into legislative decision-making and the lobbying process
British library newspapers parts I–V (historical source)	Keyword-searchable database of newspapers located throughout the British Isles	“Free-grown sugar”, ‘free-labour sugar”, “Slave-grown sugar”	Newspaper articles and commentaries. 639 results	The primary sources found here allowed us to see how sugar production and consumption were discussed during this time period. The material was rich in discussion of the sugar industry and its regulation. There were occasional references to consumer preferences and decision-making
*Times* digital archive (historical source)	Keyword-searchable database of Britain’s paper of record	“Free-grown sugar”, ‘free-labour sugar”, “Slave-grown sugar”	Newspaper articles “free-grown sugar” 54 “slave-grown sugar” 303 “free-labour sugar” 102	Similar to above
*Economist* historical archive (historical source)	Keyword-searchable database of all articles published in the *Economist,* a newspaper that covered news from a classical liberal perspective	“Free-grown sugar”, ‘free-labour sugar”, “Slave-grown sugar”	Newspapre articles, analysis and commentaries “free-grown sugar” 9 “free-labour sugar” 62 “slave-grown sugar” 31	Articles provided insight into the ways in which sugar production and consumption were discussed during this time period. They allowed us to understand why policymakers permitted slave-produced sugar to flood the British market after 1846
Slavery and anti-slavery: A transnational archive. Part I: debates over slavery and abolition Coverage: up to 1888 (historical and scholarly source)	Slavery and anti-slavery includes documents from the United States and Europe. Over 1.5 million cross-searchable pages 7,247 books, 80 serials, more than fifteen manuscript collections, & court records	“Free-grown sugar”, ‘free-labour sugar”, “Slave-grown sugar”	Books, serials, court records 9 relevant sources identified. The earliest dates from 1793, the approximate time the market category was created	Our search of this database yielded fewer relevant documents than expected but it did allow us to material than expected but this reflects the learn about popular attitudes to slavery in the 1790s, the approximate time the market category was created

As we read these secondary sources, we learned about how nineteenth century opponents of slavery advocated the consumption of free-grown sugar from British India. We also discovered that we needed to learn about additional topics, such as the history of sugarcane cultivation in Bengal and the role of Quaker merchants in anti-slavery political movements. We also learned that we needed additional information about the long-standing political rivalry between the British business interests connected to the East India Company and the so-called “West India Interest,” the British companies and individuals with commercial ties to Caribbean slavery. Additional secondary sources were then read. Based on our extensive reading of the relevant secondary sources, we were able to produce a detailed timeline (chronology) and list of historical actors related to our project. Armed with these lists, we were able to begin our research using primary sources.

In recent years, many nineteenth-century British publications have been digitized, indexed, and rendered keyword searchable. These publications include the London *Times*, which is keyword searchable back to 1785, the *Manchester Guardian*, and a range of other contemporary newspapers from communities throughout the British Isles. Using the chronology and list of names discussed above, we methodically searched these databases for newspaper articles that dealt with competition between free-grown and slave-produced sugar in the British market. Our searches of newspapers included advertisements as well as editorial content. We also searched the digitized transcripts of debates in the British parliament, which covers the period from 1803, and a database of parliamentary papers, which includes a range of government reports. Our keyword searches used the terms “slave-grown sugar” and “free-grown sugar”, the common nineteenth-century terms. We also searched for “free-labour sugar,” a term used by some contemporaries that had the same meaning as “free-grown sugar.”

Quantitative data related to the creation of the ethics-driven market category of free-grown sugar in the past is necessarily more limited than if we were observing contemporary market category formation. For instance, we lack access to market research reports that would allow us to establish, with confidence, what precise share of the sugar consumed by the UK population in, say, 1870 had been produced with slave labour. However, for part of the period covered by our study we have proxy data in the form of the UK’s annual trade statistics published. These statistical tables, which aggregated data collected at ports of entry across the British Isles, allow us to determine what proportion of the country’s sugar came from a particular country. Since the secondary literature (Davis 1999) tells us when each sugar-producing country ended slavery in its sugarcane fields, we can determine approximately what proportion of UK sugar was produced with what contemporaneous British people would have regarded as “slave labour.”

### Analytic Process

We took active steps during our analytical process to limit the extent to which these subjective biases might distort our data analysis. The literature on inductive and abductive research methods (e.g., Strang 2015), and on ideology, confirmation bias, and motivated reasoning in academic research (Tetlock 1994; Bisgaard 2015), made us conscious that ideology influences pattern matching and thus might affect our abductive research. As noted above, both of the researchers live in the social context of the United Kingdom and have left-of-centre political views that likely motivate us to discover examples of market failures in the primary sources we analysed. We were aware that researchers who have different political views (e.g., libertarians who emphasize the positive aspects of capitalism) might arrive at different conclusions after analysing the same primary sources. For instance, a libertarian might be less likely to identify cases of market failure in the primary sources listed in the tables above and more inclined to spot examples in which free markets and individuals’ pursuit of self-interest had actually helped to address the problem of slavery. As we prepared to analyse our primary sources, we were acutely aware of the need for ideological bias mitigation.

Early in our research process, we discovered a book by a U.S. libertarian academic who shares our opposition to slavery but who argues that freeing the private sector from state control helps to address the problem of slavery (Wright 2017). Wright argues that slavery is antithetical to capitalism and that if we increase the degree to which the economy corresponds to the ideal-type known as *laissez*-*faire* capitalism, slavery will be undermined. Although we did not accept all of this researcher’s findings, the book was a powerful reminder to us of how ideology influences a conscientious researcher’s interpretation of documents. Prior to reviewing the primary sources listed in the tables above, we reminded ourselves that researchers with other ideological commitments might interpret them differently. We also reminded ourselves, and each other, that we needed to try to interpret each document in an open-minded fashion consistent the mindset that researchers engaged in process tracing are expected to cultivate (Bennett 2010; Mahoney 2015). Both members of the research team have broadly similar ideological outlooks that they brought into the document analysis process. We also need to report that one of the researchers had a slightly different interpretation of the documents as a result of her birth and childhood experiences in Africa. The personal background of that researcher caused her to be more sensitive to the issue of race when analysing our primary sources and in our subsequent efforts to formulate an explanation for the decline of the market category of free-labour sugar.

We used source criticism and hermeneutics to analyse each of the primary sources we encountered. The technique of source criticism requires the researcher to ask a series of questions about each document they examine. These questions begin with the issue of whether the document is authentically historical, rather than a modern forgery, and then proceed to deeper questions such as the identity of the document’s creator, the intended reader of the document, and the ultimate purpose of the document. Since many of the texts we encountered were pamphlets and newspaper articles that discussed sugar and slavery, we sought to determine the agenda of each document’s author or authors. Hermeneutical research methods are grounded in “a theory of textual interpretation that posits that the meaning of language and texts arise through their relationship to” their context, which means that “specific texts, or parts of texts, therefore need to be understood in relationships to contexts and vice versa” (Kipping et al. 2014, p. 320). Hermeneutics thus requires the researcher to acquire deep knowledge of “the cultural, social, as well as temporal context” in which a given document was created. Without such knowledge, the researcher might misunderstand a primary source that alludes to phenomena that would have been common knowledge to contemporaries, and to specialists in the history of the period, but not to most present-day social-scientific researchers. We therefore made a point of reading widely around the topic, gaining contextual knowledge of nineteenth-century Britain that allowed the team to understand the primary sources we found.

Our abductive research process allowed us to develop a system of historical periods for understanding the evolution of the market category of free-labour sugar. In most forms of historical research, a major task of the researcher is to divide the period covered by their study into historical periods and sub-periods (Green 1995). This analytical process, which corresponds to what organizational studies scholars call *temporal bracketing* (Langley and Truax 1994; Langley 1999), is called *periodization* (Bentley 1996). Periodization allows the researcher and her readers to organize historical phenomena into coherent periods so that patterns of continuity, discontinuity, and historical causation are understandable. A researcher’s system of periodization reflects her values and understanding of historical causation. As they reviewed the primary and secondary sources related to the rise and fall of the category of free-labour sugar, the researchers developed a timeline of our topic (discussed below).

Finally, we used process tracing to arrive at causal explanations for the historical phenomena we discovered. Process tracing is an interpretative method in qualitative research that is informed by Bayesian theory. It requires the researcher who uses historical documents to test causal explanations for an *explanandum* against a graduated standard of evidence (Bennett 2008). In process tracing, researchers estimate the strength of the supporting evidence on a scale that ranges from “straw-in-the-wind” to “doubly decisive.” Depending on the nature of the supporting evidence, the researcher calibrates the degree of certainty with which they express confidence in their conclusions about the nature of the causal relationship they have arrived at based the reading of primary and secondary sources (see Table 1 in Collier 2011). Whereas quantitative researchers express degrees of certainty using confidence intervals, process tracing scholars indicate to readers their degree of confidence through linguistic markers of certainty. Methodologically influential papers that use process tracing include Tannenwald (1999)’s study of the reasons for the development of the US nuclear weapons taboo after 1945 and Schultz’s (2001) study of the Fashoda Crisis of 1898. Process tracing researchers tend to express less confidence in causal inferences about distant historical topics than on more recent phenomena because the primary sources relevant to recent periods of history are generally more abundant, permitting the researcher to be more confident of their findings about causal relationships (Mahoney 2015).

One downside of using historical research methods is that it often involves data gaps caused by the loss of primary sources over time. Awareness of such data gaps impels scholars who use process tracing methods to adjust downwards their level of confidence in their research findings. In conducting the research for this case study, we were fortunate enough to find an abundance of relevant primary sources that allow us to present many findings with a high degree of confidence. However, we also encountered an important data gap, namely the absence of primary sources in which nineteenth-century consumers recorded their precise motives for buying free-labour sugar. Another important data gap we found was the absence of internal records from Smith and Leaper, the London firm that played a crucial role in the market category of free-labour sugar. The fact the firm’s records did not survive somewhat reduces the degree of confidence with which we report that Joseph Leaper’s motivations for creating this market category were at least partially humanitarian rather instrumentalist.

### Our Case Study

Our research allowed us to develop a system of periodization for understanding the evolution of the free-labour sugar market category (see Table 2). The first of our historical periods begins in the 1600s when sizeable volumes of slave-produced sugar first entered the British market and ends in the late 1790 s, when members of Britain’s new anti-slavery movement attacked sugar as an immoral commodity. Per capita sugar consumption rose dramatically in Britain in the seventeenth and eighteenth centuries (Trentmann 2016, p. 58). There are no traces of people in this period debating whether the consumption of the products of slave labour was ethical. This period clearly corresponds to the “silencing” phase in the Arjaliès and Durand (2019) model. The emergence of the anti-slavery movement in the 1760s and 1770s led, in the late 1780s and early 1790s, to a period of “turmoil” that witnessed boycotts of sugar by so-called “anti-saccharites”, who regarded the consumption of sugar as inherently immoral. The first boycott of sugar by anti-slavery proponents took place in 1791. In 1792, entrepreneurs in London developed the market category of free-labour sugar using raw material imported from India, a nation people in London believed did not have chattel slavery. This market category reached the “inclusion” phase in the 1790 s and persisted up to the 1840s, when it disappeared and became re-silenced. Although slave-produced sugar was imported by Britain until the 1880 s, the market category itself did not re-appear.

**Table 2:** Our system of periodization for understanding the market category of free-labour sugar

Historical period	Key features	Corresponding phase in the Arjaliès and Durand (2019) model
Indifference, c. 1600 to 1770s	Per capita sugar consumption rises in Britain. Whether slavery is ethical is a topic that goes undiscussed until the 1760 s when Granville Sharp publishes first anti-slavery pamphlet	“Silencing”/“judgment silence”
1780 to 1790s: attacks on the legitimacy of sugar consumption and appearance of the market category of free-labour sugar	Anti-slavery activists attack the consumption of sugar as an inherently immoral activity. So-called “anti-saccharites” first boycott sugar in 1791 Entrepreneurs in London develop the market category of “free-labour sugar” using raw material imported from India. This sugar becomes the prototype of the market category.	“Turmoil and judgement questioning”
Market category of free-labour sugar is established, 1790s to 1830s	A segment of the British population purchases “free-labour sugar” which is more expensive that slave sugar	“Stability and judgment inclusion” Existence of category with normative attributes
1840s, market category disappearance	Slave-produced sugar floods British market after 1846 tariff changes. No traces of the existence of the market category of free-labour sugar can be found in 1850s and 1860s	Does not correspond to any of the phases in the model developed by Arjaliès and Durand Return to “Silencing”, which we term “(Re)Silencing”

### “Turmoil”: The British Sugar Trade and the Rise of Anti-slavery Sentiment

The following section shows how contestation led on to turmoil. The emergence of the anti-slavery movement was, in our view, a crucial precondition for the creation in the 1790s of the market category of free-grown sugar. The British anti-slavery movement is generally regarded to have been begun in the 1760s (Davis 1999), prior to which point British people accepted the existence of slavery as natural and unremarkable. The activists began their efforts to eliminate slavery by campaigning for the end of the trans-Atlantic slave trade, immediate freedom for any Black slaves who reached mainland Britain, and the gradual abolition of slavery in Britain’s colonies. In addition to lobbying parliament to end slavery, these social activists adopted the supplementary strategy of using consumer power to fight slavery. Britain’s first documented boycott of slave-produced sugar took place in 1791, roughly a generation after the publication of the first anti-slavery pamphlet in English. One credible estimate indicates that this campaign affected the behaviour of 400,000 consumers. Sales of sugar fell, albeit only briefly, by between a third and a half (Midgley 1996).

The boycott initially took the form of total abstention from sugar products. Those who abstained from all sugar, the so-called “anti-saccharites” included a disproportionate number of members of small religious sects that were known for eschewing most luxuries and were well outside of the cultural mainstream (Midgley 1996). Most British consumers who disliked slavery were reluctant to abstain from all sugar and were thus caught between the demands of conscience and their desire to eat sweet substances. The result was the creation of an entrepreneurial opportunity for a group of London merchants connected to the East India Company, the firm that had a statutory monopoly on trade with India.

The East India Company, which was routinely described by contemporaries as having exploited the population of India, was arguably the most controversial firm in English-speaking world (Erikson 2014). From 1788 to 1795, there was a high-profile effort in the British parliament to impeach an official of the East India Company for human rights abuses in Bengal (Bowen 2005). The 1791 boycott of West Indian sugar by opponents of African slavery created an opportunity for the East India Company to both divert attention from its activities in India and to develop a profitable side-line. In 1791, a group of warehouse owners and other merchants in London who owned East India Company shares began pressuring the firm’s leadership to import sugar from India. These individuals were supported by Britons in India who had recognized that growing demand for non-slave sugar in the British market had created an entrepreneurial opportunity. In March 1792, the company’s Court (i.e., board) of Directors approved efforts to import Indian sugar. The arguments used in the surviving internal correspondence we observed (Committee of Warehouses 1792) strongly suggests that ethical concerns related to West Indian slavery were not a motivation for any of the individuals involved in the production, transport, and wholesaling of the East Indian sugar that retailed in Britain as free-labour sugar. As we show below, some of relevant retailers, however, appear to have been motivated by such ethical concerns.

The sugar imported into the British Isles from the territories of the East India Company was produced by peasant cultivators who lived around the Bay of Bengal in present-day Bangladesh and India. Historians have shown that Enlightenment thinkers contested the definition of “slavery” and sometimes conceptualized slavery as merely one pole of a continuum of exploitation rather than a wholly distinct, isolated phenomenon (Lott 1998). However, Bengali sugar producers were generally perceived by eighteenth-century and nineteenth-century British people as falling clearly into the category of “free”. They were thus seen as being in a very different position than either the enslaved plantation workers in the British West Indies or the agricultural slaves of southern India. The earliest known text that urges consumers to buy East India sugar on anti-slavery grounds dates from February 1792, when the firm of Smith and Leaper in London’s Bishopsgate Street placed an advert in the *Morning Chronicle* newspaper.

The product advertised in the *Morning Chronicle* in early 1792 appears to have been the prototype of the market category of free-labour sugar. Unfortunately, the firm of Smith and Leaper did not leave us any internal correspondence that would allow us to begin the difficult task of ascertaining the relative importance of ethical and purely commercial considerations as motivators for their decision to sell free-grown sugar. However, given that Joseph Leaper was a philanthropist who played an important role in efforts to eradicate smallpox from the world (Royal Jennerian Society 1821, p. 33), humanitarian considerations might have been at least part of the firm’s motivation to develop this market category. Other London retailers quickly imitated Smith and Leaper’s practice of marketing East Indian sugar as the produce of free labour, with the result that sales of East Indian sugar “increased tenfold” during the 1790s (Holcomb 2016, p. 61). To use the terminology of Kovács and Hannan (2010), the ethics-driven market category created by Smith and Leaper and other merchants associated with the East India Company was a “crisp” one in that they had succeeded in distinguishing East Indian sugar, which was generally seen as the product of unambiguously free workers, from the West Indian sugars produced by slaves. In the next section, we discuss how this new market category became firmly established.

### “Inclusion”: Establishment of the Labour-Free Sugar Market Category

In the early 1800s, the market category of free-labour sugar became firmly established in London and spread to urban centres throughout the British Isles. For instance, a newspaper in Dublin published a list of Irish shopkeepers that exclusively sold sugar that was produced by free labour (Major 2012, p. 300). During this period, which corresponds to the “inclusion” phase in the Arjaliès and Durand (2019) model, the market category achieved greater public visibility, through pamphlet campaigns and the endeavours of a small number of businessmen, some of whom were passionately opposed to slavery. To promote the use of Bengali sugar within the British Isles, the East India Company later partnered with James Cropper, a Quaker merchant in Liverpool whose opposition to slavery was consistent with the teachings of the religious sect of which he was a member. Historians have long debated whether Cropper’s attacks on the use of slave-produced sugar from the West Indies were motivated primarily by his religious convictions or by simple self-interest, with the latter being the favourite view of Marxist historians (e.g., Williams 1943). A respected historian has concluded that Cropper regarded his personal self-interest and God’s interest in freeing slaves as entirely congruent (Davis 1961). Unfortunately, the surviving records of Cropper’s firm^1^ do not allow us to compare the profitability of Cropper’s free-labour sugar venture with his other business ventures, so we are unable to venture a definitive statement about whether his efforts to develop this market category were motivated more by pecuniary or moral considerations.

**Fig. 1:**
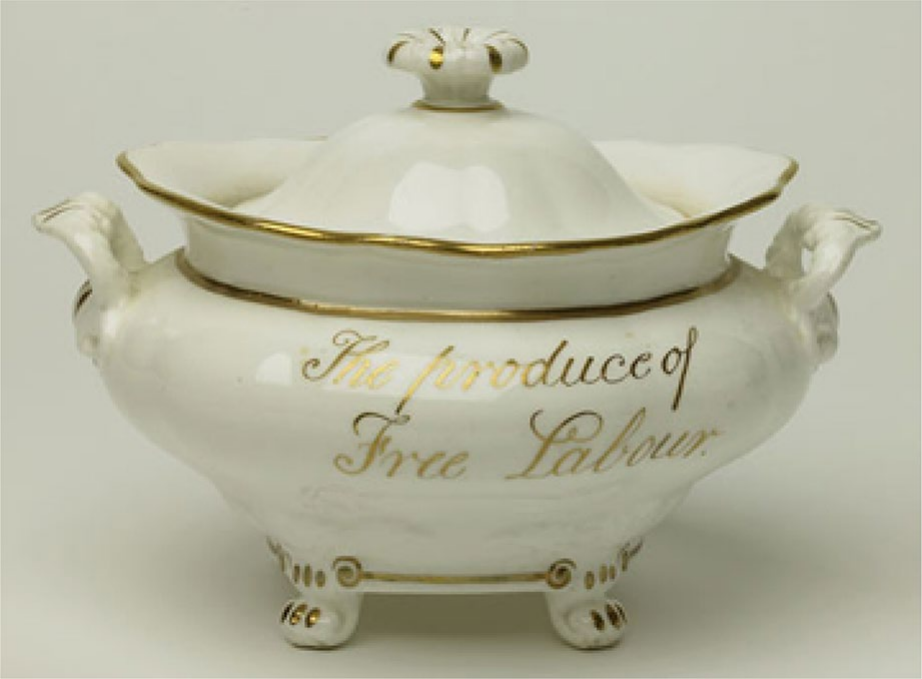
Sugar bowl inscribed ‘East India Sugar. The produce of Free Labour’. International Slavery Museum, Liverpool, Accession number MMM.1994.111. Used With Permission

Another entrepreneur who facilitated the perpetuation of the market category of free-grown sugar was the ceramics producer Josiah Wedgewood, who produced decorative sugar bowls that allowed households to inform visitors that they had behaved ethically by refusing to buy cheaper, slave-labour sugar (see Fig. 1; Guerty and Switaj 2004). As in the case of James Cropper, Wedgewood regarded his material interest and the moral cause of freeing the slaves as entirely congruent (Oldfield 2012). Since sugar in this period was not sold in branded packaging, the texts and images on Wedgewood’s sugar dishes played an important role in the development of the market category, as they allowed households to virtue signal to tea-drinking visitors.

William Naish’s 1828 pamphlet *Reasons for Using East India Sugar* contained vivid descriptions of the atrocities committed on the sugar plantations of the British West Indies (Naish 1828). Naish informed his readers that the sugar slaves are whipped with “a strongly plaited whip, called a cart whip, each lash of which makes an incision on the bank” (Naish 1828, p. 8). He reminded his readers that “if we purchase the commodity, we participate in the crime… the slave-dealer, the slave-holder, and the slave-driver are the agents of the consumer, and may be considered as employed and hired by him to procure the commodity” (Naish 1828, p. 14). Shops installed signs boasting that their sugar was produced by free labour, although the absence of either a certification regime or any laws mandating truth in advertising meant that some unscrupulous grocers likely sold slave-labour sugar as free-grown sugar (Midgley 1996; Holcomb 2014; Conrad 2018). Grocers in provincial English towns appear to have used simple, easy to remember statistics of dubious accuracy to persuade customers to purchase more expensive free-grown sugar from India: “by six families using East India sugar instead of West India sugar one slave less is required” (Stobart 2013, p. 46).

As the profile of free-labour sugar grew, firms selling slave-produced sugar from the West Indies responded by saying that their products were superior in taste and colour to free-labour sugars from Bengal. Indeed, an 1825 pamphlet that urged British consumers to continue to purchase West Indian sugars suggested that ethical considerations were the only reason any sane consumer would ever purchase Bengali sugar. This pamphlet declared that “Traders in East India Sugar, conscious that its quality, strength and price cannot give it an advantageous competition with West Indian sugar”, had been forced to rely on “an appeal to moral feeling” (Anonymous 1825 p. 8). Firms connected to the West Indian sugar trade also used the argument that Bengali sugar producers were legally unfree by blurring the lines between low-caste Hindu peasants and African chattel slaves. In effect, they attempted to render fuzzy the distinction between slave-labour sugar from the West Indies and free-labour sugar from India. The claim that Bengali peasants were serfs was rejected by James Cropper (1823) and other advocates of the use of Bengali sugar (Macaulay 1823; Christian Observer 1825) who vehemently denied that the peasantry of Bengal and the other sugar-producing regions of India were in a condition resembling slavery. In supporting the view that the peasants of Bengal were truly free farmers, these authors invoked the author of Britain’s leading experts on Indian society, such as the Sanskritologist Henry Thomas Colebrooke. For instance, Zachary Macaulay (1823, p. 91) answered the rhetorical question “but in Bengal is not sugar cultivated by slaves?” with “certainly not. In proof of this, I confidently appeal to Mr. Colebrooke and every other authority on the subject who is worthy of credit.” Another abolitionist published that the “husbandmen” of Bengal “are of free condition and where praedial slavery at all exists within the Company’s territories, the servile labourers are not the subjects of leases, but are the absolute property of the landholders, for whose benefit they work, and who hold their lands in perpetuity” (Stephen 1825, pp. 103–104).

Faced with the attempt by the West Indian sugar producers to make the boundaries of the market category fuzzy, Cropper and others who sold Bengali sugar published documents intended to ensure that the boundaries of the free-labour sugar category remained crisp. Due a combination of higher costs and the inability of Bengali sugar refiners to produce sugars as pure as those of the plantations of the West Indies, free-grown sugar never captured more than a small share of the British market (Amin 1984; Attwood 1985; Ratledge 2004). However, Major (2012 p. 301) has observed that whilst East India sugar never seriously challenged West India dominance of the UK sweetener market, free-grown sugar from India was “invested with important symbolic value” during the final years of the campaign to abolish slavery in the West Indies. We now shift to a discussion of re-silencing and the demise of this market category.

### “(Re)Silencing”: Market Category Disappearance

In this period, the market category disappeared due to re-silencing. The phaseout of slavery in the British West Indies in 1834-8 was followed by approximately a decade in which British consumers were not called upon to make an ethical choice between the free-grown and slave-labour sugar, since steep protective tariffs effectively excluded slave sugar from the British market (Ratledge 2009). From 1834 to 1846, the UK’s tariff system distinguished between British colonial producers of sugar from sugar from those countries that still had slavery, such as the United States, Cuba, and Brazil. Differential customs duties were put in place so that West Indian sugar planters with paid workforces would not have to compete with sugar planters whose governments had not chosen to abolish slavery. In 1846, the British government controversially decided to equalize the duties on British colonial and foreign (i.e., slave-grown) sugar (Green 1991, p. 232; Schuyler 1918, p. 72). The government did so on the grounds that allowing cheaper slave-produced sugar into the British market would be good for consumers (Huzzey 2010).

The equalization of the sugar duties was swiftly followed by a surge in the proportion of the UK’s sugar that had been produced using slave labour. From the 1840s up until the 1880s, when Brazil and Cuba at last abolished slavery, significant quantities of slave-produced sugars entered British ports (UK Parliament 1868a; Curtin 1954, see also Table 3). In 1868, Cuba, a country in which the phaseout of slavery did not begin until the 1870s, was the UK’s single most important source of raw sugar. Brazil, an important sugar exporter, had slavery until 1888. In the 1870s, the UK began import more European beet-derived sugar, but it continued to consume significant quantities of tropical sugar that had been produced by slaves. As late as 1880, the tonnage of slave-produced tropical sugars arriving in British ports still represented 12% of all imports (UK Parliament 1880). British newspapers in this period frequently referred to the country’s reliance on slave labour for its sugar (e.g., The Pall Mall Gazette 1876; The Ipswich Journal 1877; Glasgow Herald 1883; Manchester Courier 1886). Speakers in parliament discussed the country’s imports of slave-produced sugar in a fashion that suggested that this fact was common knowledge (Buxton 1850; Baring 1857; Davis 1862, p. 172; Courtney 1879, p. 181; Hill 1879, p. 121). In view of this evidence, we can conclude that sugar consumers of this period, at least those who read newspapers, would have been aware that they were consuming the products of slave labour.

**Table 3:** Countries identified by government statisticians as Sources of sugar, by order of importance, in period 1864-8 (UK Parliament 1868b)

Type of sugar	Source countries	Nature of sugar production
Refined sugars	Holland	Temperate, free-labour country
	Belgium	Temperate, free-labour country
Unrefined, white clayed sugar	Cuba	Tropical, slave
	Mauritius	Tropical, free labour
	British India	Tropical, free labour
Brown clayed sugar and yellow muscovado	Cuba	Tropical, slave
	Puerto Rico	Tropical, largely non-slave
	Mauritius	Tropical, free labour
	British India	Tropical, free labour
	British West Indies	Tropical, free labour
Brown muscavado	Philippine Islands	Tropical, largely non-slave
	Cuba	Tropical, slave
	Brazil	Tropical, slave
	Mauritius	Tropical, free labour
	British India	Tropical, free labour
	British West Indies	Tropical, free labour
Molasses	Cuba	Tropical, slave
	British West Indies	Tropical, free labour

One might have thought that the post-1846 surge in imports of slave-produced raw sugar would have prompted the re-creation of the market category of free-grown sugar rather than re-silencing. However, we have found no evidence of the market category’s existence despite our diligent searches of digitized newspapers and other primary sources. Moreover, when the UK government conducted a detailed study of the UK’s sugar trade in 1862, none of the importers, refiners, wholesalers, and retailers who testified before parliament said anything that suggested that such a market category existed or that UK consumers of this generation were concerned about whether their sugar was the product of slave labour or free labour. The 1862 investigation resulted in a richly detailed report about the UK sugar market. This report describes how retailers offered consumers a wide variety of sugar products differentiated by colour, texture, and degree of saccharine content. Many market categories within the broad category of “sugar” thus existed. However, the report provides no evidence that retailers, even the so-called “west-end grocers” who served the affluent, sold sugar products that were categorized by whether they were produced with free as opposed to slave labour. None of the wholesalers who testified before the committee suggested that anti-slavery or other ethical considerations influenced the behaviour of importers, retailers, or consumers (Travers 1862, p. 18).

British sugar consumers in the period from the 1840s to the 1880s appear to have been indifferent to the moral issue of whether their sugar was produced by slave or free labour. This indifference should not be attributed to lack of knowledge of the provenance of their sugar, since newspapers and other sources referred to the fact much of the country’s raw sugar was produced by slaves in Cuba and Brazil. The knowledge that British sugar consumers were supporting slavery with their purchases did not create an outcry similar that seen in the 1780s and 1790s, when growing opposition to slavery had contributed to the development of the market category of free-labour sugar. In the next section, we provide a multi-factor explanation for the non-existence of this market category in the post-1840 period.

We now venture an explanation for why the ethics-driven market category of free-grown sugar disappeared after 1840. As we reviewed our primary sources and generated our system of historical periodization (Table 2) we discovered that the market category of free-labour sugar had disappeared around 1840. We then sought to formulate various explanations for its disappearance. We developed our explanation for the disappearance of the market category using process tracing, an analytical procedure described above, and found evidence that corresponds in strength to the “hoop” standard. Within process tracing, the hoop standard is stronger than merely indicative “straw-in-the-wind” evidence but less strong than “smoking gun” evidence (Collier 2011). For this reason, we adopt an intermediate level of confidence in expressing our explanation for why the market category disappeared and thus use somewhat tentative language. We also signal our moderate degree of confidence in our explanation by referring to other possible causal explanations. It should be noted that our degree of confidence in the accuracy of the causal inferences presented below is somewhat limited by the fact that our evidence base of nineteenth-century documents is less comprehensive than a data set about a more recent historical phenomenon would likely be.

The first possible explanation for the disappearance of the category after 1840 that occurred to us was “compassion fatigue”, a phenomenon that is familiar to both social entrepreneurs and organization studies scholars today (Simpson et al. 2014). However, the more we thought about this possible explanation for the decline of the market category, the less persuasive simple compassion fatigue seemed to us as a sufficient explanation, as historians have documented instances of ethics-driven business ventures in nineteenth-century Britain that persisted for longer periods of time and did not fade away because of compassion fatigue. The ethics-driven businesses that were apparently immune to compassion fatigue included social enterprises that supplied high-quality social housing to working-class Britons at below market rates (Birch and Gardner 1981; Maltby and Rutterford 2016). These ventures, some of which are still in existence today (e.g., the Peabody Trust) had one thing in common: their beneficiaries were white Britons in the United Kingdom rather than people of different races overseas.

In our view, changing attitudes towards race are the key cultural-cognitive variable that helps to explain why so-called compassion fatigue affected the market category of free-labour sugar when it did not affect other ethics-driven market categories. A leading historian of the anti-slavery movement in nineteenth-century Britain (Hall 2002) has argued that a form of compassion fatigue developed in the middle of the century as an effect of the hardening of racial ideologies. According to her research, after slavery had been extirpated within the British Empire in the 1830s, British popular opinion tired of the subject of slavery and turned to other social causes aimed at improving the lives of working-class white Britons. The diminishing interest in the condition of Black workers in the New World was connected to a well-documented shift in British attitudes towards race after the 1830s and 1840s. The intensification of anti-Black racism Britain was associated with the advent of pseudo-scientific doctrines that caused many British people to doubt the very humanity of Black people. From the 1830s onwards, many British people were attracted to the theory of polygenesis, which rejected the Biblical teaching that all humans were descended from a single couple and instead taught that each of the five races of man had evolved separately from primates (Knapman 2016).

Polygenesis and the perversion of Darwin’s theory of evolution (1859) appears to have caused many British people to be less concerned about the rights and interests of non-white individuals (Lorimer 2009). During the 1860s, many of Britain’s most influential intellectuals, including Charles Dickens, expressed virulently racist views in the course of defending Governor Eyre, a military officer who had massacred Black people in Jamaica. According to a historian who has studied the Eyre Controversy, the intensely anti-Black views expressed by these thinkers would have been regarded as unacceptably illiberal a generation earlier, during the high-water mark of the anti-slavery movement (Levy 2002). The growing tendency in British culture from the mid-1800s onwards to dehumanize Black people helps to explain the decline of the market category after 1840. If racial identities were more salient to the average British consumer in 1865 than they had been to his or her parents in the 1830s, the resulting change in consumer cognition would help to explain why the market category of free-labour sugar disappeared. We have therefore concluded that shifting attitudes to race probably contributed to the decline of the market category.

In analysing our texts, we arrived at another, and not mutually exclusive, possible explanation for the decline of the market category of free-labour sugar. This explanation relates to the population of entrepreneurs who developed and sustained the market category. Our explanation for the formation of the market category centres on the emergence in the 1790s of significant numbers of British consumers willing to pay a premium for free-labour sugar, which created the possibility of a new market category. However, demand-side pressures are insufficient by themselves to explain the creation of this market category, as the market category would not have been created without the efforts of a small number of entrepreneurs such as Joseph Leaper and James Cropper. The latter was a sincere and passionate opponent of slavery. The sudden death of Cropper in 1840 appears to have had a significant impact on the market category. The firm founded by James Cropper survived his death, but his immediate successors do not appear to have shared his interest in sugar. In 1845, James Cropper Junior effected a dramatic change in the strategy of the family firm by pivoting into paper production, the industry in which James Cropper plc now operates (Cropper 2004). The effects on the market category of the death of James Cropper Senior are a reminder of role of historical contingency in market category evolution.

## Discussion and Implications

On the basis of the discussion above, we are able to identify the paper’s five main theoretical contributions. First, our paper historicizes and problematizes the concept of “slavery” by showing that its boundaries were contested by actors in the past. In the 1820s, British people argued about which sugar producers were actually free, with representatives of the slave-owning Caribbean sugar producers advancing the self-interested argument that peasants of Bengali were the equivalent of slaves. Such arguments were intended to render fuzzy the perceived difference between slave-labour and free-grown sugar, thereby undermining the latter market category. Opponents of slavery who advocated the consumption of free-grown sugar from India stressed that the differences in the legal status of Bengali and Caribbean sugar producers were substantial. The implication for the present of this historical finding is that we might expect actors involved in debates about modern slavery to engage in similar work in contesting the definition and boundaries of slavery, expanding and contracting the category of “the enslaved” to suit their purposes. Our finding that definitions of slavery have been contested since the era of the first social movements against slavery should, we hope, inspire further research in management on the conceptual history of the term “slavery” itself.

A second lesson modern slavery scholars should derive from our historical research is that consumer indifference, as opposed to simple ignorance (lack of knowledge on the part of consumers) may be the major barrier to getting consumers to act against slavery. As noted above, Christ and Burritt (2018) found that the “ignorance” of consumers about the provenance of products is an important barriers to the elimination of modern slavery in the supply chains of Australian firms. Throughout the nineteenth century, British people were aware that much of the sugar arriving in the country was the product of slave labour. In the early nineteenth century, a sufficient number of consumers were upset about the issue of slavery as to support the market category of free-labour sugar. After 1840, the market category disappears even though consumers would have known that sugar was arriving from countries that still had slave labour. The implication of this finding for researchers and policymakers interested in combatting slavery in the present is that simply informing consumers and other decision-makers that there is slave labour in the supply chain may not be enough to prompt them into action. The challenge in translating knowledge into action may be particularly acute if there are forces in the wider culture, such as a morally parochial indifference to the suffering of foreigners, that can contribute to consumer indifference.

Third, we have argued above that intensifying racism after 1840 contributed to consumer indifference about whether their sugar was produced by slaves. We presented evidence that a number of trends in British culture contributed to the growing perception in Britain that people of African descent were inferior and almost a separate biological species. In effect, the de-humanization and Otherization of African people contributed to the demise of the market category of free-labour sugar by encouraging consumer indifference to the question of the sugar had been produced by slaves. Although supply chains can, in theory, link consumers to slaves who are of the same race and ethnic origins, slavery, whether historic or in the present, often involves the exploitation of individuals who are racially or ethnically different than the beneficiaries of their labour. Modern slavery scholars may wish to consider whether the rise of xenophobic, racist, or nationalist modes of thought may make it more difficult to get consumers to be concerned about the issue of slavery and then act on those concerns.

The fourth major contribution of our paper will be of interest to both consumption ethics scholars and modern slavery researchers. As we pointed out in our literature review, the research in management on consumer ethics is informed by a historical metanarrative that holds that there is a natural tendency for each generation of consumers to become more interested in ethical issues. This optimistic historical metanarrative links technological progress and rising living standards to consumers’ increasing interest in ethics. Our historical research calls into question the theory of linear moral progress that influences how many business ethics researchers understand consumption ethics. As we show, the market category of free-grown sugar disappeared from the British market after several decades of existence. In the 1780s and 1790s, anti-slavery activists made the consumption of sugar an ethical question in British culture for the first time. After 1792, a proportion of British sugar buyers insisted that the sugar served in their households had to be unconnected to slavery. After the 1840s, however, the commodity became demoralized and a (re)silencing phase began. The market category of free-grown sugar did not re-appear in the period between 1846 and 1880, which suggests that few if any consumers of this era had moral qualms about consuming sugar produced by slaves, even though sources of free-grown sugar were readily available.

The disappearance of the market category of free-labour sugar is remarkable when we remember that the UK’s GDP per capita rose dramatically over the course of the nineteenth century. In 1801, the earliest year in our period for which GDP per capita estimates are available, UK GDP per capita was US$2,300 in 2017 values, which implies that living standards in the UK were similar to those in present-day Uganda, Benin, and Zimbabwe. Despite the poverty of the average British person in this era, the country had developed the market category of free-grown sugar. By 1860, UK GDP per capita was $3,900, a considerable improvement over the 1801 level, yet the market category had disappeared. Up until the 1880s, the UK imported large quantities of slave-grown sugar. The average British person in 1880 was considerably better off than they had been in 1820 yet consumers in the latter years did not display the same willingness to pay more for free-grown sugar that many in a comparatively poor generation had displayed in the 1820s. The relationship between increasing living standards and consumer concern about the ethics of using the products of slave labour would therefore appear to be non-linear, challenging some existing work on ethical consumption (i.e., Barnett et al. 2005). As we noted in our literature review, many business ethics scholars appear to associate rising levels of consumer interest in ethical consumption with affluence. However, as this case study suggests, rising GDP per capita does not necessarily translate into more ethical consumers, which is a significant contribution to on-going debates around the ‘attitude-behaviour’ gap (Boulstridge and Carrigan 2000; Auger and Devinney 2007; Caruana et al. 2016).

Our paper shows that it is possible for consumers’ level of interest in a particular ethical issue to decline rather than to rise with the passage of time. Our case study is thus inconsistent with the rather optimistic historical metanarrative that is present in much management and ethical consumption research. The optimistic historical metanarrative problematized by our paper is just one example of a hitherto unexamined historical metanarrative that has informed how management researchers understand the world. The use of historical metanarratives, which are stories about human history that allow individuals to make sense of data points by constructing narratives, is widespread in social-scientific research. The researchers who use historical metanarratives to understand the world are frequently unaware that they are doing so (Butters 2017). For this reason, we strongly suspect that there are other historical metanarratives that inform management research and which have yet to be critically examined. Other scholars may therefore wish to use historical research to examine and critique the other historical metanarratives that have hitherto informed management research.

Fifth, and finally, our study reminds Modern slavery scholars of the utility of incorporating historical research into their analysis of the present-day problem of slavery. As we noted above, much of the literature on Modern slavery is decidedly ahistorical and ignores the parallels and continuities with historical forms of slavery, such as African chattel slavery in the plantation complex of the New World, with Modern slavery. We believe that theoretically-informed historical research on historical slavery similar to that presented in this paper has the capacity of help us as researchers to understand the problem of modern slavery and to then provide guidance to policymakers, social activists, and others who are seeking to develop effective solutions to this problem.

### Implications for Public Policy

Can we count on the voluntary efforts of ethically motivated consumers and entrepreneurs to eliminate modern slavery from global supply chains? Or is state action the only effective way to eliminate modern slavery? One lesson we could draw from our study is that ethics-driven market categories have a very limited capacity to effect social change. There is little doubt that the British consumers who paid a premium for free-labour sugar believed that the cumulative effect of such ethical purchases by millions of individuals would be freedom for many enslaved African individuals in the sugar-producing regions of the world. However, there is little evidence that it had a significant impact on the bottom lines of West Indian plantations and the other British firms that profited from slavery.

Slavery within the British Empire was ended by state action (an Act of Parliament) rather than the voluntary efforts of the British consumers who purchased free-labour sugar. By 1890, slavery had disappeared from all of the regions in the New World that supplied Britain’s sugar and in every case slavery was terminated by state action, not consumer sentiment working through market forces. Our research thus suggests that while participating in the creation of ethics-driven market categories may help at the margin to address present-day ethical issues in business, such as modern slavery, campaigning for state action may be more effective. This finding is relevant to debates around the effectiveness of current legislation covering modern slavery. Some national governments, such as the UK and Australia, have engaged in a process of ‘outsourcing governance’ (Mayer and Phillips 2017) whereby the state delegates a variety of governance functions and authority to private actors most often large firms. Our research, which has revealed the limits of private action as a tool for eliminating slavery, suggests that outsourcing governance is unlikely to be effective and that policymakers should instead use a different approach to tackle modern slavery.

## Conclusion

Slavery remains a pressing social issue that justly occupies the attention of a range of policymakers and academics. As Hathaway (2008, p. 7) reminds us, “the fight against slavery is one of the very few human rights imperatives that attracts no principled dissent”. It is, therefore, vital for researchers to develop an accurate and comprehensive understanding of the forces that influence attitudes towards unfree labour. The dynamics that influenced the sugar market in nineteenth-century Britain, which included long overseas supply chains, unstable levels of consumer interest in the underlying ethical issues, and fluctuations in the salience of national and racial identities continue to be relevant to understanding how consumers think about slavery. Our study of the rise and fall of this market category has, we hoped, demonstrated the utility of historical research to scholars of business ethics and anti-slavery activists. Sugar was not, of course, the only commodity whose history is intimately intertwined with that of slavery. We believe that by engaging more with the historical relationship between slavery and capitalism, management scholars will be able to make other theoretical contributions.

## Footnotes

1 The surviving records, which are kept in the Liverpool Maritime Museum archive, were investigated by one of the authors.
